# Biomechanical modulation of collagen fragment-induced anabolic and catabolic activities in chondrocyte/agarose constructs

**DOI:** 10.1186/ar3009

**Published:** 2010-05-12

**Authors:** Tina T Chowdhury, Ronny M Schulz, Sonpreet S Rai, Christian B Thuemmler, Nico Wuestneck, Augustinus Bader, Gene A Homandberg

**Affiliations:** 1 School of Engineering and Materials Science, Queen Mary University of London, Mile End Road, London, E1 4NS, UK; 2 Department of Cell Techniques and Applied Stem Cell Biology, University of Leipzig, Deutscher Platz 5, Leipzig, 04103, Germany; 3 Department of Biochemistry and Molecular Biology, University of North Dakota School of Medicine and Health Sciences, Box 9037, Grand Forks, ND 58202, USA

## Abstract

**Introduction:**

The present study examined the effect of collagen fragments on anabolic and catabolic activities by chondrocyte/agarose constructs subjected to dynamic compression.

**Methods:**

Constructs were cultured under free-swelling conditions or subjected to continuous and intermittent compression regimes, in the presence of the N-terminal (NT) and C-terminal (CT) telopeptides derived from collagen type II and/or 1400 W (inhibits inducible nitric oxide synthase (iNOS)). The anabolic and catabolic activities were compared to the amino-terminal fibronectin fragment (NH_2_-FN-f) and assessed as follows: nitric oxide (NO) release and sulphated glycosaminoglycan (sGAG) content were quantified using biochemical assays. Tumour necrosis factor-α (TNFα) and interleukin-1β (IL-1β) release were measured by ELISA. Gene expression of matrix metalloproteinase-3 (MMP-3), matrix metalloproteinase-13 (MMP-13), collagen type II and fibronectin were assessed by real-time quantitative polymerase chain reaction (qPCR). Two-way ANOVA and the *post hoc *Bonferroni-corrected *t*-test was used to examine data.

**Results:**

The presence of the NT or CT peptides caused a moderate to strong dose-dependent stimulation of NO, TNFα and IL-1β production and inhibition of sGAG content. In some instances, high concentrations of telopeptides were just as potent in stimulating catabolic activities when compared to NH_2_-FN-f. Depending on the concentration and type of fragment, the increased levels of NO and cytokines were inhibited with 1400 W, resulting in the restoration of sGAG content. Depending on the duration and type of compression regime employed, stimulation with compression or incubation with 1400 W or a combination of both, inhibited telopeptide or NH_2_-FN-f induced NO release and cytokine production and enhanced sGAG content. All fragments induced MMP-3 and MMP-13 expression in a time-dependent manner. This effect was reversed with compression and/or 1400 W resulting in the restoration of sGAG content and induction of collagen type II and fibronectin expression.

**Conclusions:**

Collagen fragments containing the N- and C-terminal telopeptides have dose-dependent catabolic activities similar to fibronectin fragments and increase the production of NO, cytokines and MMPs. Catabolic activities were downregulated by dynamic compression or by the presence of the iNOS inhibitor, linking reparative activities by both types of stimuli. Future investigations which examine the signalling cascades of chondrocytes in response to matrix fragments with mechanical influences may provide useful information for early osteoarthritis treatments.

## Introduction

The ability of degradation products of the extracellular matrix to regulate cartilage homeostasis and influence osteoarthritis (OA) disease progression has been extensively studied [1,2]. For instance, different types of matrix fragments derived from fibronectin or collagen can signal and amplify catabolic processes in chondrocytes that act to either remove tissue components for repair or to initiate reparative signals [3,4]. Chondrocytes will additionally respond to biomechanical perturbation such that mechanical loading on normal or diseased tissue will contribute to signalling cascades and upregulate synthetic activity or increase the levels of inflammatory mediators [5-7]. Our understanding of what factors initiate the early phase of matrix damage in OA is poor. The question of whether mechanical loading modulates matrix fragment induced mechanisms for repair and/or degradation in early stage OA is not known.

The inflammatory pathways induced by fibronectin fragments (FN-fs) in chondrocytes are well characterised [8,9]. For instance, the amino-terminal fibronectin fragment (NH_2_-FN-f) has potent catabolic activities and was shown to increase cytokines (interleukin-1α (IL-1α), interleukin-1β (IL-1β), tumour necrosis factor-α (TNFα), interleukin-6 (IL-6)), matrix metalloproteinases (matrix metalloproteinase-3 (MMP-3), matrix metalloproteinase-13 (MMP-13)) and nitric oxide (NO) production in human and bovine cartilage [10-14]. The signalling pathways involve the mitogen activated protein kinase (MAPK) and nuclear factor-kappa B (NFκ B) cascades mediated by stimulation of integrin receptors, leading to a suppression of proteoglycan synthesis and increased proteoglycan depletion in chondrocytes [15-19]. In addition, the N-terminal (NT) telopeptide from collagen type II was shown to upregulate MMP-3 and MMP-13 levels in human and bovine cartilage [20-22]. However, collagen fragments (Col-fs) containing the NT or C-terminal (CT) telopeptide regions were much slower at increasing MMP levels when compared to the NH_2_-FN-f [23]. This difference could be reflected in the differential rate of activation of members of the MAPK or NFκB family, leading to the production of common catabolic mediators such as NO [19]. Recently, we showed that compressive loading inhibits NH_2_-FN-f induced NO production and restores matrix synthesis in chondrocytes cultured in agarose constructs [24]. It is plausible that mechanical loading competes with the catabolic pathways induced by the matrix fragments and contributes to early reparative signals in chondrocytes. The present study therefore compared the effect of Col-fs with the NH_2_-FN-f on the production of NO, cytokines and MMPs in chondrocyte/agarose constructs subjected to dynamic compression.

## Materials and methods

### Chondrocyte isolation and culture in agarose constructs

Articular cartilage was harvested from the porcine metacarpalphalangeal joints of freshly slaughtered 12-month-old pigs from a local abattoir (FEL GmbH, Leipzig, Germany). Cartilage tissue was pooled from six joints, diced and incubated on rollers for one hour at 37°C in Dulbecco's Modified Eagle's Medium (DMEM) supplemented with 10% (v/v) foetal calf serum (FCS) + 2 μ M L-glutamine, 5 μ g.ml^-1 ^penicillin, 5 μ g.ml^-1 ^streptomycin, 20 mM Hepes buffer, and 0.05 mg/ml L-ascorbic acid + 700 unit.ml^-1 ^pronase, and incubated for a further 16 hours at 37°C in DMEM + 10% FCS (all from Sigma-Aldrich, Taufkirchen, Germany) supplemented with 2 mg.ml^-1 ^collagenase A (Biochrom KG, Berlin, Germany). The cell suspension was washed and viable chondrocytes counted using a haemocytometer and trypan blue. Cells were finally resuspended in medium at a cell concentration of 8 × 10^6 ^cells.ml^-1 ^using established methods [25,26]. Briefly, the cell suspension was added to an equal volume of molten 6% (w/v) agarose type VII in Earle's Balanced Salt Solutions (EBSS) to yield a final cell concentration of 4 × 10^6 ^cells.ml^-1 ^in 3% (w/v) agarose (Sigma-Aldrich, Taufkirchen, Germany). The chondrocyte/agarose suspension was transferred into a sterile stainless steel mould, containing holes 10 mm in diameter and 3 mm in height and allowed to gel at 4°C for 20 minutes to yield cylindrical constructs. All constructs were maintained in culture in 1 ml of DMEM + 10% FCS at 37°C in 5% CO_2 _for 24 hours.

### Dose-response effect of telopeptides in chondrocyte/agarose constructs

The dose-response effect of the N-terminal (NT) and C-terminal (CT) telopeptides derived from collagen type II were examined in constructs cultured under free-swelling conditions for 48 hours. The synthetic peptides were less than 10 kDa in size and were synthesised by Sigma Genosys (Haverhill, UK), using sequences published previously [20-23]. More specifically, the NT peptide corresponds to the amino-terminal region of collagen type II and contains 19 amino acids (residues 182 to 212) with an additional four glycine-proline-hydroxyproline (GPX) tripeptide repeat resulting in a short 31-mer peptide (sequence: QMAGGFDEKAGGAGLGVMQGPMGPMGPRGPP). The CT peptide corresponds to the carboxyl-terminal end of collagen type II and contains 24 amino acids (residues 1218 to 1241; sequence: IDMSAFAGLGPREKGPDPLQYMRA). The constructs were cultured in 1 ml of DMEM + 1 × ITS liquid media (Sigma-Aldrich, Taufkirchen, Germany) supplemented with either 0, 0.05, 0.5, 5 and 50 μM NT or 0.05, 0.5, 5 and 50 μM CT peptide in the presence and absence of 1 mM N-(3-(aminomethyl) benzyl)acetamidine.2HCL (1400 W) (Merck Biosciences, Nottingham, UK). 1400 W is a chemical inhibitor which specifically inhibits the inducible nitric oxide synthase (iNOS) enzyme. An optimal concentration of the scrambled form of the NT (SN (19 residues; sequence: GPGAGQPGKGRGPAPLQFGMAMMDMADPGEV)) and CT (SC (24 residues; sequence: MARFPAMLGPARDPISYQKEGDGL)) peptides were used as negative controls (both at 50 μM). A commercially available 30 kDa NH_2_-FN-f at 1 μM was used as a positive control (Sigma-Aldrich, Poole, UK). At the end of the culture period, the constructs and corresponding media were immediately stored at -20°C prior to biochemical analysis.

### Application of dynamic compression

**Figure 1:**
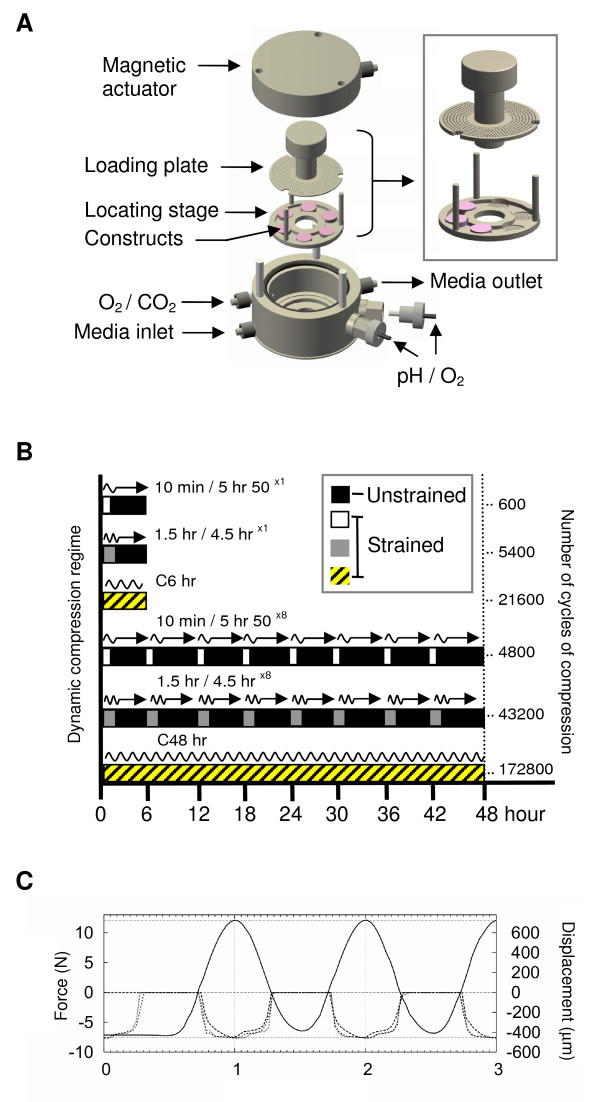
**Schematic illustrating components of the bioreactor device and experimental compression regimes**. **(a) **Six chondrocyte/agarose constructs were held under confined conditions in a locating stage as shown in the inset. Both the locating stage and loading plate were fluid permeable and perforated to facilitate nutrient transport to all surfaces of the construct. **(b) **The compression regimes are shown in the middle panel resulting in a total number of cycles ranging from 600 to 172,800 over a 6- or 48-hour culture period. Black bars indicate unstrained periods equivalent to 0% strain. **(c) **Black lines show typical response profiles for load and displacement generated with a sinusoidal waveform at time = 0 (dash), 1 (dash dot) and 48 hours (square dot), respectively.

The present study utilized a bioreactor device (Ingenieurburo, GmbH, Braunschweig, Germany) to apply compressive loading to chondrocyte/agarose constructs, using a system described previously [27]. Briefly, the bioreactor vessel consists of two chambers with a cylindrical lid that fits a magnetic actuator connected to a stainless steel loading plate (Figure 1a). Six constructs were held under confined conditions in a locating stage with an inner and outer wall (Figure 1a, inset). This arrangement limits axial movement of the loading plate therefore allowing the system to apply a known compressive strain. Both the locating stage and loading plate were fluid permeable (TECAPEEK, Ensinger GmbH and Co., Nufringen, Germany) and perforated to facilitate nutrient transport to all surfaces of the construct. The lower chamber has two ports enabling media and gas exchange while the upper chamber fits two connectors for pH and O_2 _biosensors. Culture media containing either 50 μM NT or 50 μM CT or 1 μM NH_2_-FN-f and/or 1 mM 1400 W were introduced to the lower chamber. The vertical motion of the magnetic actuator and loading plate was controlled by a magnet field induced by an external Tesla NdFeB magnet which rotated above the bioreactor. Various continuous and intermittent compression regimes were employed over a 6 or 48 hour culture period resulting in a total number of compression cycles which ranged from 4800 to 172800 (Figure 1b). The following periods of compression were applied to constructs in a dynamic manner at 15% strain and a frequency of 1 Hz: 10 minutes compression with a 5 hour 50 minutes unstrained period (10 minutes/5 hr 50^×1^); 1.5 hour compression with a 4.5 hour unstrained period (1.5 hr/4.5 hr^×1^); 6 hours of continuous compression (C6); 10 minutes compression with a 5 hour 50 minute unstrained period repeated 8 × (10 minutes/5 hr 50^×8^); 1.5 hour compression with a 4.5 hour unstrained period repeated 8 × (1.5 hr/4.5 hr^×8^) and 48 hours of continuous compression (C48). Dynamic compression was applied with a load and displacement control feedback system. A typical response for the load and displacement profile generated with a sinusoidal waveform is illustrated in Figure 1c. This ensured a maximum load of 12 N which remained constant during the 48 hour compression period. The displacement curves showed similar profiles at time = 0, 1 and 48 hours and was equivalent to a deformation of 450 μM and displacement of 15% strain. For control constructs, the fluid permeable loading plate was situated 0.8 mm above the construct to facilitate nutrient transport and cultured in an unstrained state at 0% strain for the same time period within the bioreactor device. At the end of the culture period, all constructs and corresponding media were immediately stored at -80°C prior to analysis.

### RNA isolation, cDNA synthesis and real-time quantitative polymerase chain reaction (qPCR)

RNA was isolated from chondrocytes cultured in agarose using protocols described in the QIAquick^® ^Spin gel extraction and RNeasy^® ^kits, as previously described [24,28]. (Qiagen, Hilden, Germany). Following the manufacturer's instructions, Ambion's DNA-*free *DNase treatment and removal reagents were used to eliminate any contaminating DNA from the RNA sample (Ambion, Applied Biosystems, Warrington, UK). RNA was quantified on the Nanodrop ND-1000 spectrophotometer (LabTech, East Sussex, UK) and reverse transcription performed using manufacturer's protocols from the M-MLV First-Strand cDNA synthesis kit, oligo(dT)_15 _primer and a total of 200 ng of RNA (Promega, Manheim, Germany). For real-time quantitative PCR, the cDNA was amplified in 25 μl reaction mixtures containing 1 μl cDNA, 12.5 μl SYBR^® ^Green PCR Master Mix, primer pairs (Table 1) and nuclease free PCR grade water (Applied Biosystems) using an automated PCR robot (CAS-1200™, Corbett Research, Cambridge, UK). Each sample was run in duplicate on the 72-well thermal system of the Rotor-Gene™ 3000 instrument (Corbett Research). Thermocycling conditions comprised of an initial polymerase activation step at 95°C for 3 minutes, followed by 35 cycles at 95°C for 30 s, at 55°C for 60 s and at 72°C for 60 s. Following amplification, a melt curve was obtained to ensure no detection of primer-dimers and non-specific products. In order to screen for contamination of reagents or false amplification, PCR controls were prepared for each sample by preparing identical reaction mixtures except for the addition of the template (NTC). No reverse transcriptase (NoRT) controls were additionally included in each PCR assay.

**Table 1:** Description of the sequences used to quantify gene expression and real-time reaction efficiencies of PCR assays

Gene	Gene ID	Sequences	Product size (bp)	Efficiency
**MMP-3**				
	396769	*Forward: *5'-ACCCAAGAAGTATCCACACCCT-3'	215	1.98 ± 0.06
		*Reverse: *5'-TGCTTCAAAGACAGCATCCACT-3'		
**MMP-13**				
	397346	*Forward: *5'-CCAAAGGCTACAACTTGTTTCTTG-3'	77	1.99 ± 0.03
		*Reverse: *5'-TGGGTCCTTGGAGTGGTCAA-3'		
**Collagen type II**				
	397323	*Forward: *5'-CGCTGAACATCCTCACAAC-3'	249	1.98 ± 0.19
		*Reverse: *5'-TCCTGTAGATACGCCTAAGC-3'		
**Fibronectin**				
	397620	*Forward: *5'-GACAGATGAGCTTCCCCAAC-3'	752	2.02 ± 0.09
		*Reverse: *5'-CACTGCCAAAGCCTAAGCAC-3'		
**GAPDH**				
	396823	*Forward: *5'-AATCCCATCACCATCTTCCA-3'	318	2.03 ± 0.01
		*Reverse: *5'-TGTGGTCATGAGTCCTTCCA-3'		

Primers used in quantitative polymerase chain reaction (qPCR) experiments with SYBR green produced amplicons of 77 to 752 base pairs with efficiency values between 1.98 and 2.03. GAPDH, glyceraldehyde 3-phosphate dehydrogenase; MMP-3, matrix metalloproteinase-3; MMP-13, matrix metalloproteinase-13.

Fluorescence data were collected during the annealing stage of amplification and data were analysed using the RG-3000™ qPCR software (version 6, Corbett Research). Baselines and thresholds were automatically set by the RG-3000™ qPCR software and used after manual inspection. The cycle threshold (C_t_) value for each duplicate reaction was expressed as the mean value and the results were exported into Microsoft Excel for further analysis. The data obtained by PCR assay for Glyceraldehyde-3-Phosphate Dehydrogenase (GAPDH) were validated as a reference gene by displaying the C_t_ values as Box and Whisker plots and the distribution examined under mechanical loading conditions (data not shown). The C_t _values for GAPDH remained stable with no changes detected under all treatment conditions, suggesting its suitability as a reference gene. Relative quantification of MMP-3, MMP-13, collagen type II and fibronectin signals was accomplished by normalizing each target to the reference gene, GAPDH and to the calibrator sample (unstrained, untreated sample) by a comparative C_t _approach [29]. For each sample, the ratio of target ΔCt and reference ΔCt was calculated, as shown in equation 1.(1)

Where: E represents the efficiencies obtained for the target and reference gene. ΔCt_target _represents the difference in C_t _values for the mean calibrator or sample for the target gene. ΔCt_Reference_ represents the difference in C_t _values for the mean calibrator or sample for the reference gene, GAPDH.

PCR efficiencies for primer pairs with SYBR green were derived from standard curves (n = 3) by preparing a 10-fold serial dilution of cDNA from a sample which represents the untreated sample. The real-time PCR efficiencies (E) of amplification for each target was defined according to the relationship, E = 10 [-1/slope]. The R^2 ^value of the standard curve exceeded 0.9998 and revealed efficiency values presented in Table 1.

### Biochemical analysis

At the end of the experiment, constructs were digested in phosphate buffered saline (PBS) supplemented with 10 mM L-cysteine and 10 mM EDTA, pH 6.5 for 60 minutes at 70°C and subsequently incubated with 1.66 Units/mL agarase for 16 hours at 37°C and with 0.1 units/mL Papain for 1 hour at 60°C, as previously described [25,26]. DNA levels were determined in the agarase/papain digests by Quant-iT™ PicoGreen^® ^dsDNA assay according to manufacturer's instructions (Molecular Probes, Eugene, OR, USA). Sulphated glycosaminoglycan (sGAG) content was determined using the 1, 9-dimethyl-methylene blue dye-binding assay in agarose/papain digests and media samples and the values normalized to DNA levels [25,26]. The production of NO was determined in media by converting nitrate to nitrite using 1 unit.ml^-1 ^nitrate reductase in 40 μM NAPDH, 500 μM glucose 6-phosphate, 160 unit.ml^-1 ^glucose 6-phosphate dehydrogenase and 20 mM Tris-HCL for 15 minutes at 37°C and total nitrite assayed spectrophotometrically at 540 nm using the Griess reaction, as described previously [30,31]. The levels of IL-1β and TNFα were determined in media samples by commercial ELISA kits according to manufacturer's instructions (R & D Systems Europe Ltd, Abingdon, UK).

### Statistics

For the dose-response studies, data represent the mean and standard error of the mean (SEM) values of six replicates from two separate experiments. For the mechanical loading experiments, data represent the mean and SEM values of eight replicates from two separate experiments. Statistical analysis was performed by a two-way analysis of variance (ANOVA) and the multiple *post hoc *Bonferroni-corrected *t*-tests to compare differences between treatment groups as indicated in the figure legend. In all cases, a level of 5% was considered statistically significant (*P *< 0.05).

## Results

### Telopeptides increase NO production and inhibit sGAG content in a dose-dependent manner

**Figure 2:**
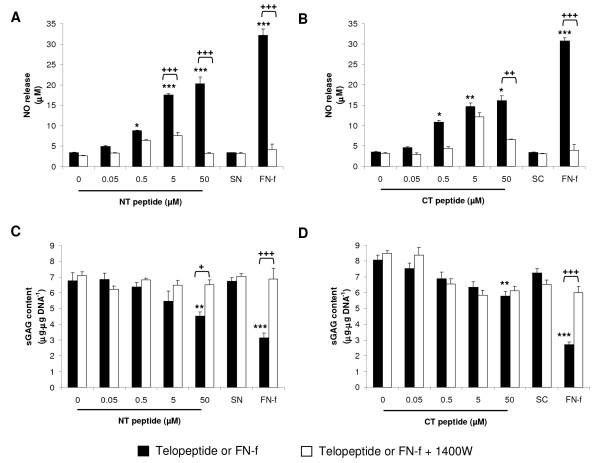
**Dose-response effect of NT and CT telopeptides**. Constructs were cultured with NT (0.05 to 50 μM) or CT (0.05 to 50 μM) peptides under free-swelling conditions in the presence or absence of 1 mM 1400 W for 48 hours: **(a) **NO release and **(b) **sGAG content (n = 6). A scrambled form of the NT (SN) and CT (SC) peptide were used as negative controls (both at 50 μM). An NH_2_-FN-f (1 μM) was used as a positive control. (*) indicates significant comparisons for 0 vs fragment; (+) indicates significant comparisons for fragment vs fragment + 1400 W (n = 6 ±).

The ability of NT and CT peptides to influence NO release and sGAG content in constructs cultured for 48 hours are illustrated in Figure 2. The levels of NO were enhanced by the presence of the NT or CT peptides, with significant levels at 0.5 μM and increasing up to 50 μM when compared to untreated controls (*P *< 0.001 and *P *< 0.05; Figure 2a and 2b, respectively). This effect was similar to treatment with NH_2_-FN-f and showed significant levels of NO production when compared to untreated controls (*P *< 0.001). At 50 μM, co-incubation with 1400 W inhibited telopeptide or FN-f-induced NO release with levels returning to basal values. In contrast, the presence of the NT or CT peptides did not influence sGAG content at a concentration ranging from 0.05 to 5 μM when compared to untreated controls (Figure 2c, d). At 50 μM, the presence of the NT or CT peptides partially inhibited sGAG content (*P *< 0.01) and this effect was reversed with 1400 W for the NT peptide, only (*P *< 0.05; Figure 2c). The NH_2_-FN-f strongly inhibited sGAG content (*P *< 0.001) and the response was reversed with 1400 W (*P *< 0.001). The control SN or SC peptides did not significantly influence NO production and sGAG content in the presence and absence of 1400 W.

### Telopeptides increase cytokine levels in a dose-dependent manner

**Figure 3:**
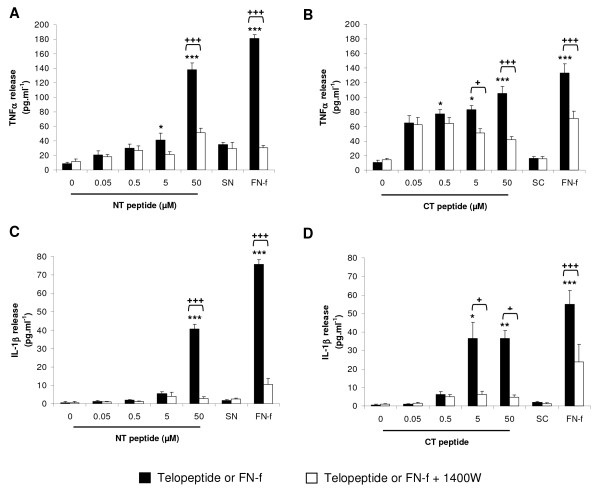
**Dose-response effect of NT and CT telopeptides**. Constructs were cultured with NT (0.05 to 50 μM) or CT (0.05 to 50 μM) peptides under free-swelling conditions in the presence or absence of 1 mM 1400 W for 48 hours: **(a) **TNFα release and **(b) **IL-1β (n = 6). A scrambled form of the NT (SN) and CT (SC) peptide were used as negative controls (both at 50 μM). An NH_2_-FN-f (1 μM) was used as a positive control. (*) indicates significant comparisons for 0 vs fragment; (+) indicates significant comparisons for fragment vs fragment + 1400 W (n = 6).

We next characterised the dose-response effect of NT and CT peptides on the production of IL-1β and TNFα in constructs cultured for 48 hours (Figure 3). The presence of the NT or CT peptides enhanced TNFα release when compared to untreated controls, with significant levels at 5 (*P *< 0.05) and 50 μM (*P *< 0.001) for the NT peptide and at 0.05 (*P *< 0.05), 5 (*P *< 0.05) and 50 μM (*P *< 0.001) for the CT peptide (Figure 3a and 3b, respectively). At 50 μM, peptide-induced TNF-α release was inhibited with 1400W (*P *< 0.001; Figure 3a, b). The presence of the NT or CT peptides increased IL-1β production in a concentration-dependent manner (Figure 3c and 3d, respectively). This effect was inhibited with 1400 W resulting in a significant reduction at 50 μM NT (*P *< 0.001) or with 5 and 50 μM CT peptide (both *P *< 0.05). The presence of the NH_2_-FN-f increased maximal levels of TNFα and IL-1β production when compared to untreated controls and this effect was inhibited with 1400 W (*P *< 0.001). The control SN and SC peptides did not influence cytokine levels in the presence and absence of 1400 W.

### Dynamic compression modulates telopeptide induced NO release and restores sGAG content

**Figure 4:**
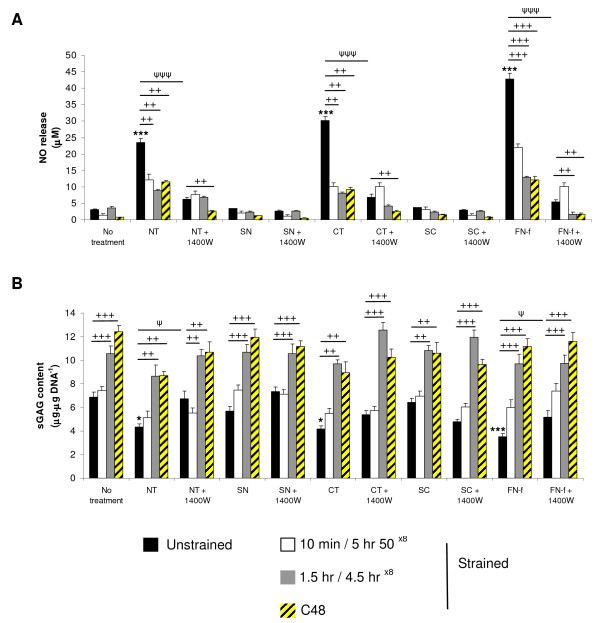
**Effect of NT and CT telopeptides and dynamic compression (15%, 1 Hz) on NO release (a) and sGAG content (b)**. Unstrained and strained constructs were cultured with 50 μM NT or CT peptide and/or 1 mM 1400 W for 48 hours (n = 8). SN and SC peptides (50 μM) were used as negative controls. NH_2_-FN-f (1 μM) was used as a positive control. (*) indicates significant comparisons in unstrained constructs for no treatment vs fragment; (ψ) indicates significant comparisons in unstrained constructs for fragment vs fragment + 1400 W; + *P *< 0.05, ++ *P *< 0.01, +++ *P *< 0.001 indicates significant comparisons between treatment conditions as shown (n = 6).

Having demonstrated that treatment with NT or CT peptides influenced NO release and sGAG production in a concentration-dependent manner, subsequent studies examined the effect of continuous or intermittent compression on the peptide or NH_2_-FN-f induced response (Figure 4). Under no treatment conditions, no significant differences were detected for NO release in unstrained constructs and constructs subjected to compression for 10 minutes/5 hr 50^×8^, 1.5 hr/4.5 hr^×8 ^or C48 hours (Figure 4a). In unstrained constructs, the presence of the NT or CT peptides enhanced NO levels when compared to constructs cultured without the peptide (both *P *< 0.001). Stimulation with compression for 10 minutes/5 hr 50^×8^, 1.5 hr/4.5 hr^×8 ^or C48 hours (all *P *< 0.01), or incubation with 1400 W inhibited NO release (*P *< 0.001). This effect could be further downregulated by co-stimulation with both compression for C48 hours and 1400 W in peptide treated constructs (*P *< 0.01). In unstrained constructs, the NH_2_-FN-f increased maximal levels of NO release when compared to untreated controls (*P *< 0.001). This effect was inhibited under all compression regimes or culture with the iNOS inhibitor (all *P *< 0.001). Co-stimulation with both compression for 1.5 hr/4.5 hr^×8 ^or C48 hours and 1400 W abolished FN-f-induced NO release with values returning to basal levels (both *P *< 0.01).

Under no treatment conditions, sGAG content was enhanced following stimulation with intermittent compression for 1.5 hr/4.5 hr 50^×8 ^or with continuous compression for C48 hours (both *P *< 0.001; Figure 4b). In unstrained constructs, the presence of the NT or CT peptides inhibited sGAG content (both *P *< 0.05) and this effect was partially reversed with compression for 1.5 hr/4.5 hr^×8 ^or C48 hours and/or 1400 W. In unstrained constructs, the NH_2_-FN-f inhibited sGAG content when compared to untreated constructs (*P *< 0.001). This effect was reversed with compression for 1.5 hr/4.5 hr^×8 ^or C48 hours and/or culture with the iNOS inhibitor (all *P *< 0.001). We did not detect significant changes in NO release and sGAG content in constructs cultured with the control SN or SC peptides and/or 1400 W.

### Dynamic compression inhibits telopeptide induced cytokine levels

**Figure 5:**
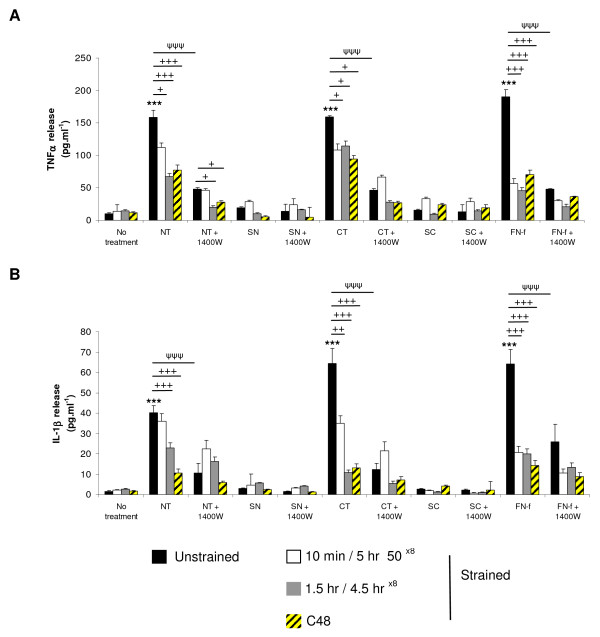
**Effect of NT and CT telopeptides and dynamic compression (15%, 1 Hz) on cytokine production**. Unstrained and strained constructs were cultured with NT or CT peptide (both 50 μM) and/or 1400 W (1 mM) for 48 hours: **(a) **TNFα release and **(b) **IL-1β release (n = 8). SN and SC peptides (50 μM) were used as negative controls. NH_2_-FN-f (1 μM) was used as a positive control. (*) indicates significant comparisons in unstrained constructs for no treatment vs fragment; (ψ) indicates significant comparisons in unstrained constructs for fragment vs fragment + 1400 W; + *P *< 0.05, ++ *P *< 0.01, +++ *P *< 0.001 indicates significant comparisons between treatment conditions as shown (n = 8).

Figure 5 examined the effect of continuous and intermittent compression on cytokine production in the presence and absence of telopeptides or NH_2_-FN-f for 48 hours. Under no treatment conditions, the levels of TNFα or IL-1β were not significantly influenced by compression for 10 minutes/5 hr 50^×8^, 1.5 hr/4.5 hr^×8 ^or C48 hours. In unstrained constructs, the presence of NT or CT peptides increased TNFα and IL-1β production (both *P *< 0.001). This response was broadly inhibited under all compression regimes and/or culture with 1400 W. In unstrained constructs, the presence of the NH_2_-FN-f increased TNFα or IL-1β release. This effect was inhibited under all compression regimes and/or 1400 W. We did not detect any significant changes in cytokine levels for constructs cultured with the control SN and SC peptides.

### Dynamic compression modulates telopeptide induced gene expression

**Figure 6:**
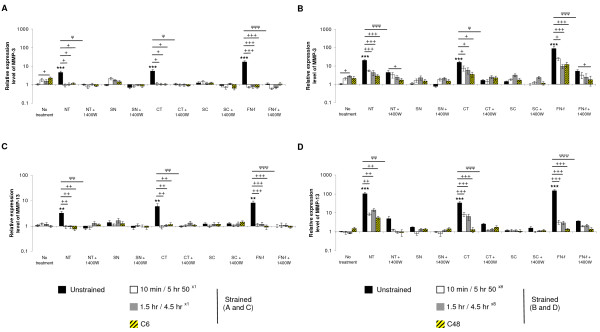
**Effect of NT and CT telopeptides and dynamic compression (15%, 1 Hz) on gene expression**. Unstrained and strained constructs were cultured with either NT or CT peptide (both 50 μM) and/or 1400 W (1 mM) for 6 or 48 hours, respectively: **(a **and **b) **MMP-3 **(c **and **d) **and MMP-13 (n = 8). SN and SC peptides (50 μM) were used as negative controls. NH_2_-FN-f (1 μM) was used as a positive control. (*) indicates significant comparisons in unstrained constructs for no treatment vs fragment; (ψ) indicates significant comparisons in unstrained constructs for fragment vs fragment + 1400 W; + *P *< 0.05, ++ *P *< 0.01, +++ *P *< 0.001 indicates significant comparisons between treatment conditions as shown (n = 8).

**Figure 7:**
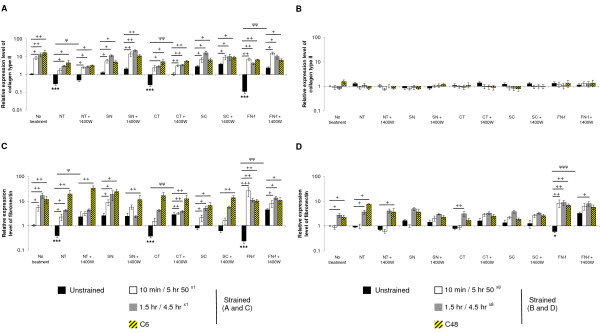
**Effect of NT and CT telopeptides and dynamic compression (15%, 1 Hz) on gene expression**. Unstrained and strained constructs were cultured with either NT or CT peptide (both 50 μM) and/or 1400 W (1 mM) for 6 or 48 hours, respectively: **(a **and **b) **collagen type II and **(c **and **d) **fibronectin (n = 8). SN and SC peptides (50 μM) were used as negative controls. NH_2_-FN-f (1 μM) was used as a positive control. (*) indicates significant comparisons in unstrained constructs for no treatment vs fragment; (ψ) indicates significant comparisons in unstrained constructs for fragment vs fragment + 1400 W; + *P *< 0.05, ++ *P *< 0.01, +++ *P *< 0.001 indicates significant comparisons between treatment conditions as shown (n = 8).

To investigate the temporal expression profile of catabolic (MMP-3, MMP-13; Figure 6) and anabolic genes (collagen type II, fibronectin; Figure 7), constructs were subjected to continuous and intermittent compression in the presence and absence of the NT and CT peptides or NH_2_-FN-f for 6 or 48 hours. At six hours, the C6 regime maximally increased MMP-3 expression when compared to unstrained constructs (*P *< 0.05; Figure 6a). At 48 hours, compression for 1.5 hr/4.5 hr^×8 ^was the only regime which increased MMP-3 expression (*P *< 0.05; Figure 6b). In unstrained constructs, treatment with telopeptides or NH_2_-FN-f for 6 or 48 hours increased MMP-3 expression (all *P *< 0.001; Figure 6a and 6b, respectively). This effect was inhibited under all compression regimes and/or culture with the iNOS inhibitor. Under no treatment conditions, compression for C6 or C48 hours did not significantly influence MMP-13 expression (Figure 6c, d). In unstrained constructs, the presence of the telopeptides or NH_2_-FN-f increased MMP-13 at 6 hours (all *P *< 0.01; Figure 6c) with maximal stimulation at 48 hours (all *P *< 0.001; Figure 6d). This effect was inhibited under all compression regimes and/or 1400 W at 6 or 48 hours. The control SN and SC peptides did not significantly influence MMP-3 or MMP-13 expression in constructs subjected to dynamic compression.

Under no treatment conditions, compression for 10 minutes/5 hr 50^×1^, 1.5 hr/4.5 hr^×1 ^or C6 hours increased collagen type II and fibronectin expression (Figure 7a and 7c, respectively). In unstrained constructs, telopeptides or NH_2_-FN-f decreased collagen type II and fibronectin expression at six hours (both *P *< 0.001; Figure 7a, c). This effect was partially reversed under all compression regimes and/or 1400 W for peptide or fragment treated constructs. At specific compression regimes, the iNOS inhibitor increased fibronectin expression in the presence of the NT peptide and FN-f at 48 hours (Figure 7d). We did not detect any significant changes in collagen type II or fibronectin expression under all test conditions at 48 hours (Figure 7b and 7d, respectively). The only exception was with the NH_2_-FN-f which increased fibronectin expression in unstrained constructs and was partially reversed by all compression regimes (Figure 7d).

## Discussion

OA is a complex disease and involves both biochemical and mechanical factors which influence disease progression. The primary causative factors are due to an increase in the levels of inflammatory mediators which contribute to an imbalance between anabolic and catabolic signalling processes. There is evidence demonstrating that the enhanced levels of FN-fs and Col-fs will initiate matrix destruction and accelerate production of catabolic mediators [1-3,32-36]. Despite advances in our understanding of the role of matrix fragments in cartilage biology, few research groups have examined whether mechanical signals could interfere with the fragment-induced pathways and modulate cell function through a positive feedback loop. In addition, pharmacological treatments have attempted to manipulate the inflammatory pathways during late stage OA [37,38]. Efforts have been largely disappointing due to lack of studies identifying the molecular/mechanical signals which control matrix repair and/or degradation in early disease states. Our understanding of the early mechanopathophysiology is poor, particularly in terms of reliable biomarkers. Thus, studies which investigate factors for early treatments and preserve the biomechanical function of the joint are both worthwhile and necessary.

The present study characterised the effect of Col-fs containing the NT or CT telopeptide regions on catabolic and anabolic activities and compared the response to the NH_2_-FN-f utilising the chondrocyte/agarose model. In dose-response studies, 48 hours of treatment with the NT or CT peptides increased NO production, enhanced IL-1β and TNFα production and inhibited sGAG content in a concentration-dependent manner. Gene expression of MMP-3 and MMP-13 was detected at 6 hours with maximal induction at 48 hours in unstrained constructs treated with telopeptides or the NH_2_-FN-f. We provide evidence that in some instances, a high concentration of NT or CT peptide (50 μM) was just as effective in stimulating catabolic activities as the NH_2_-FN-f (1 μM). The concentrations used in the present study are comparable to previous work which reported fragments encompassing the amino-terminal 29 kDa FN-f vary in concentration between 0.1 and 1 μM in human OA synovial fluids and NT and CT peptides were found to be 3 μm in a rabbit OA model [16,39-41]. We used a telopeptide concentration which represents a diseased state, so it is highly likely we are seeing maximum catabolic effects by the Col-fs. In addition, the iNOS inhibitor blocked fragment-induced catabolic activities and stimulated sGAG content. Col-fs may therefore serve to increase catabolic activities through common pathways involving NO which results in the subsequent production of MMPs and cytokines. Our findings are supported by previous studies which demonstrate the induction of MMPs and cytokines in chondrocyte monolayers and cartilage explants cultured with collagen derived peptides [20-23]. Furthermore, human chondrocytes treated with type II collagen caused sequential induction of MMPs (MMP-1, 3, 13 and 14) and cytokine production (IL-1β, IL-6, IL-8) followed by release of Col-fs from mature collagen fibres [42]. The catabolic process involved activation of p38 MAPK and NFκB leading to the production of MMPs and cytokines [42]. Overproduction of the matrix degrading enzymes will additionally release telopeptides from the triple helix, generating further Col-fs [43]. In OA, the enhanced action of MMPs on matrix proteins will generate several types of fragments released through different enzymatic pathways. It is plausible that fragments from collagen or fibronectin may arise from the degenerating matrix and promote a catabolic state through activation of common pathways. This is turn generates more matrix fragments which amplifies MMPs in a positive feedback loop [9,12]. A key question is whether a threshold concentration of fragmented matrix proteins exists, such that low levels initiate early reparative events and switch to catabolic insults at a later stage when the concentration increases above a certain level. These points raise important questions which should be investigated further.

We next examined whether dynamic compression could modulate the catabolic response induced by the fragments in constructs cultured in a bioreactor. We compared a continuous compression regime (C6 or C48) with an intermittent protocol repeated once (10 minutes/5 hr 50^×1^, 1.5 hr/4.5 hr^×1^) or eight times (10 minutes/5 hr 50^×8^, 1.5 hr/4.5 hr^×8^) during a 6 or 48 hour culture period. Our findings indicate that continuous compression was just as effective in downregulating fragment-induced NO release as intermittent compression and the response could be further downregulated with the iNOS inhibitor. In contrast, modulation of MMPs and cytokines by mechanical loading were dependent on the length and type of compression regime applied. More specifically, increasing the total number of compression cycles from 4800 to 172800 clearly had a greater inhibitory effect on cytokine production and MMP-3 and 13 expression in constructs stimulated with telopeptides. This is in contrast to constructs cultured with NH_2_-FN-f where the shortest duration of intermittent compression was just as effective in inhibiting cytokine production and MMP expression when compared to longer cycles. Our findings indicate that co-stimulation with dynamic compression and the iNOS inhibitor was marginally better at downregulating catabolic activities for some of the fragment conditions examined. The opposite effect was found for anabolic activities, such that longer periods of compression resulted in a greater magnitude of stimulation of sGAG content and expression of collagen type II and fibronectin even in the presence of fragments. The importance of these findings emphasises the nature of the mechanical stimulus in controlling catabolic and anabolic activities in chondrocytes. In a previous study utilising the cell/agarose model, the number of cycles of compression, applied in a continuous or intermittent manner was shown to be important in determining the nature of the chondrocytes metabolic response [44]. For instance, frequent bursts of intermittent compression for longer time periods favoured proteoglycan synthesis whereas shorter bursts of intermittent compression tended to favour cell proliferation. A similar response was described by other research groups which showed that the biochemical response was dependent on the duration and type of compression regime employed [45-48]. We could therefore speculate that the intermittent loading regime used in the present study mimics the physiological loading environment of cartilage. In contrast, the continuous compression regime could be interpreted as an excessive or injurious response since these conditions favoured rapid matrix turnover [49,50].

In healthy tissue, a small proportion of collagen and fibronectin levels will maintain normal matrix turnover and be released due to proteolytic digestion by MMPs [49]. In the present study, some of the enhanced collagen type II and fibronectin synthesis by dynamic compression could be an initial response at repair which may later give rise to catabolic activities involving increased synthesis of MMPs and released fragments. There is good evidence that mechanical loading conditions that mimic injury and overloading which contribute to altered patterns of load can accelerate mild damage with an early rebuilding phase by increasing MMPs and metabolic activity [51-55]. It is conceivable that the rebuilding phase may occur indirectly through the effect of altered patterns of mechanical loading by increasing the production of growth factors (TGFβ1, IGF-1, bFGF), anti-inflammatory cytokines (IL-4) or soluble mediators (Substance P, glutamate) [56-60]. Furthermore, there is evidence that integrins serve as receptors for both mechanical loading and matrix fragments implicating overlapping pathways for these signals [15,19,60,61]. Integrin-mediated mechanotransduction will contribute to chondroprotective events resulting in the cells' attempt to stimulate anabolic processes locally and assist in tissue remodelling [60]. This response will at least, in part, be dependent on the type of mechanical loading regime, its duration and whether loading was applied during the early or late stage of the disease process. Thus, conditions such as obesity or trauma that represent excessive or injurious loading will increase catabolic activities and accelerate matrix damage [48,62,63]. This disruption of matrix composition will contribute to abnormal biomechanics thereby increasing NO production, reactive oxygen species and chondrocyte death *in vivo *[64,65].

In summary, the present study demonstrates that mechanical loading modulates the catabolic and anabolic response of chondrocytes stimulated with collagen and FN-fs. The catabolic response was dependent on the concentration and type of fragment such that for conditions which represent cartilage degradation, collagen telopeptides were just as potent in increasing catabolic activities as the FN-fs. Mechanical loading could reverse the catabolic process induced by the fragments and enhance anabolic activities. However, the response was dependent on the length and type of compression regime applied. Furthermore, co-stimulation by dynamic compression in the presence of the iNOS inhibitor led to further time-dependent increases in the expression of matrix proteins and downregulation of cytokines and MMPs, implicating NO dependent pathways. The ability of chondrocytes to interact with matrix fragments and respond to biomechanical signals may be a key initiating event in the disease process. Further studies are needed to examine the complexity of the sequence of signalling events which interplay with biomechanical and matrix fragment signals for early OA therapeutic intervention.

## Conclusions

Telopeptides have dose-dependent catabolic activities similar to FN-fs and increase the production of NO, cytokines and MMPs. Catabolic activities were inhibited by dynamic compression or by the presence of the iNOS inhibitor, linking reparative activities by both types of stimuli. Future investigations which examine the signalling cascades of chondrocytes in response to matrix fragments with mechanical influences may provide useful information for early OA treatments.

## Abbreviations

Col-f: collagen fragment; C_t_, cycle threshold; CT: C-terminal telopeptide; DMEM: Dulbecco's Modified Eagle's Medium; EBSS: foetal calf serum; FCS: foetal calf serum; FN-f: fibronectin fragment; iNOS: inducible nitric oxide synthase; IL-1β: interleukin-1β; MAPK: mitogen activated protein kinase; MMPs: matrix metalloproteinases; NFκB: nuclear factor-kappa B; NO: nitric oxide; NoRT: no reverse transcriptase; NT: N-terminal telopeptide; OA: osteoarthritis; qPCR: quantitative polymerase chain reaction; sGAG: sulphated glycosaminoglycan; TNFα: tumour necrosis factor-α.

## Competing interests

The authors declare that they have no competing interests.

## Authors' contributions

TC supervised RS, SR, CT and NW who carried out experiments and analysis. TC and GH participated in the experimental design, data analysis and drafted the manuscript. All authors read and approved the final manuscript.

## References

[B1] Homandberg GA Malemud CJ Potential regulation of cartilage metabolism in osteoarthritis by fibronectin fragments Fundamental Pathways in Osteoarthritis Front Biosci 1997 713 730 10.2741/homandberg 10525477

[B2] Yasuda T Cartilage destruction by matrix degradation products Mod Rheumatol 2006 16 197 205 10.1007/s10165-006-0490-6 16906368 PMC2780665

[B3] Homandberg GA Ding L Guo D Extracellular matrix fragments as regulators of cartilage metabolism in health and disease Curr Rheumatol Rev 2007 3 183 196 10.2174/157339707781387590

[B4] Lorenzo P Bayliss MT Heinegard D Altered patterns and synthesis of extracellular matrix macromolecules in early OA Matrix Biol 2004 23 381 391 10.1016/j.matbio.2004.07.007 15533759

[B5] Griffin TM Guilak F The role of mechanical loading in the onset and progression of osteoarthritis Exerc Sport Sci Rev 2005 33 195 200 10.1097/00003677-200510000-00008 16239837

[B6] Guilak F Fermor B Keefe FJ Kraus VB Olson SA Pisetsky DS Setton LA Weinberg JB The role of biomechanics and inflammation in cartilage repair and injury Clin Orthop Relat Res 2004 17 26 10.1097/01.blo.0000131233.83640.91 15232421

[B7] Loeser RF Molecular mechanisms of cartilage destruction: mechanics, inflammatory mediators, and aging collide Arthritis Rheum 2006 54 1357 1360 10.1002/art.21813 16645963 PMC1774815

[B8] Xie D Hui F Homandberg GA Fibronectin fragments alter matrix protein synthesis in cartilage tissue cultured in vitro Arch Biochem Biophys 1993 307 110 118 10.1006/abbi.1993.1568 8239647

[B9] Xie D Hui F Homandberg GA Cartilage chondrolysis by fibronectin fragments is associated with release of several proteinases: stromelysin plays a major role in chondrolysis Arch Biochem Biophys 1994 311 205 212 10.1006/abbi.1994.1228 8203882

[B10] Homandberg GA Hui F Association of proteoglycan degradation with catabolic cytokine and stromelysin release from cartilage cultured with fibronectin fragments Arch Biochem Biophys 1996 334 325 331 10.1006/abbi.1996.0461 8900407

[B11] Homandberg GA Hui F Wen C Purple C Bewsey K Koepp H Huch K Harris A Fibronectin-fragment-induced cartilage chondrolysis is associated with release of catabolic cytokines Biochem J 1997 321 751 757 9032463 10.1042/bj3210751 PMC1218132

[B12] Homandberg GA Wen C Hui F Cartilage damaging activities of the fibronectin fragments derived from cartilage and synovial fluid Osteoarthritis Cartilage 1998 6 231 244 10.1053/joca.1998.0116 9876392

[B13] Gemba T Valbracht J Alsalmeh S Lotz M Focal adhesion kinase and mitogen-activated protein kinases are involved in chondrocyte activation by the 29-kDa amino-terminal fibronectin fragment J Biol Chem 2002 277 907 911 10.1074/jbc.M109690200 11677248

[B14] Pichika R Homandberg GA Fibronectin fragments elevate nitric oxide (NO) and inducible NO synthetase (iNOS) levels in bovine cartilage and iNOS inhibitors block fibronectin fragment mediated damage and promote repair Inflamm Res 2004 53 405 412 10.1007/s00011-004-1279-8 15316672

[B15] Forsyth CB Pulai J Loeser RF Fibronectin fragments and blocking antibodies to alpha2beta1 and alpha5beta1 integrins stimulate mitogen-activated protein kinase signaling and increase collagenase 3 (matrix metalloproteinase 13) production by human articular chondrocytes Arthritis Rheum 2002 46 2368 2376 10.1002/art.10502 12355484

[B16] Homandberg GA Meyers R Xie D Fibronectin fragments cause chondrolysis of bovine articular cartilage slices in culture J Biol Chem 1992 267 3597 3604 1740411

[B17] Yasuda T Kakinuma T Julovi SM Yoshida M Hiramitsu T Akiyoshi M Nakamura T COOH-terminal heparin-binding fibronectin fragment induces nitric oxide production in rheumatoid cartilage through CD44 Rheumatology 2004 43 1116 1120 10.1093/rheumatology/keh274 15213332

[B18] Pulai JI Chen H Im HJ Kumar S Hanning C Hegde PS Loeser RF NF-kappa B mediates the stimulation of cytokine and chemokine expression by human articular chondrocytes in response to fibronectin fragments J Immunol 2005 174 5781 5788 15843581 10.4049/jimmunol.174.9.5781 PMC2903737

[B19] Ding L Guo D Homandberg GA Fibronectin fragments mediate matrix metalloproteinase upregulation and cartilage damage through proline rich tyrosine kinase 2, c-src, NF-kappaB and protein kinase Cdelta Osteoarthritis Cartilage 2009 17 1385 1392 10.1016/j.joca.2009.03.024 19409294

[B20] Lucic D Mollenhauer J Kilpatrick KE Cole AA N-telopeptide of type II collagen interacts with annexin V on human chondrocytes Connect Tissue Res 2003 44 225 239 14660093

[B21] Jennings L Wu L King KB Hämmerle H Cs-Szabo G Mollenhauer J The effects of collagen fragments on the extracellular matrix metabolism of bovine and human chondrocytes Connect Tissue Res 2001 42 71 86 10.3109/03008200109014250 11696990

[B22] Fichter M Körner U Schömburg J Jennings L Cole AA Mollenhauer J Collagen degradation products modulate matrix metalloproteinase expression in cultured articular chondrocytes J Orthop Res 2006 24 63 70 10.1002/jor.20001 16419970

[B23] Guo D Ding L Homandberg GA Telopeptides of type II collagen upregulate proteinases and damage cartilage but are less effective than highly active fibronectin fragments Inflamm Res 2009 58 161 169 10.1007/s00011-009-8090-5 19190855

[B24] Raveenthiran SP Chowdhury TT Dynamic compression inhibits fibronectin fragment induced iNOS and COX-2 expression in chondrocyte/agarose constructs Biomech Model Mechanobiol 2009 8 273 283 10.1007/s10237-008-0134-1 18677626

[B25] Lee DA Bader DL Compressive strains at physiological frequencies influence the metabolism of chondrocytes seeded in agarose J Orthop Res 1997 15 181 188 10.1002/jor.1100150205 9167619

[B26] Lee DA Knight MM Mechanical loading of chondrocytes embedded in 3D constructs: in vitro methods for assessment of morphological and metabolic response to compressive strain Methods Mol Med 2004 100 307 324 15280603 10.1385/1-59259-810-2:307

[B27] Schulz RM Wüstneck N van Donkelaar CC Shelton JC Bader A Development and validation of a novel bioreactor system for load- and perfusion-controlled tissue engineering of chondrocyte-constructs Biotechnol Bioeng 2008 101 714 728 10.1002/bit.21955 18814291

[B28] Lee DA Brand J Salter DM Akanji OO Chowdhury TT Quantification of mRNA using real-time PCR and western blot analysis of MAPK events in chondrocyte/agarose constructs Methods Mol Med 2010 in press 10.1007/978-1-60761-984-0_6 21042967

[B29] Pfaffl MW Horgan GW Dempfle L Relative expression software tool (REST) for group wise comparison and statistical analysis of relative expression results in real time PCR Nucleic Acids Res 2002 30 e36 10.1093/nar/30.9.e36 11972351 PMC113859

[B30] Chowdhury TT Bader DL Lee DA Dynamic compression inhibits the synthesis of nitric oxide and PGE_2 _by IL-1β stimulated chondrocytes cultured in agarose constructs Biochem Biophys Res Commun 2001 285 1168 1174 10.1006/bbrc.2001.5311 11478777

[B31] Chowdhury TT Bader DL Lee DA Dynamic compression counteracts IL-1β induced release of nitric oxide and PGE _2 _by superficial zone chondrocytes cultured in agarose constructs Osteoarthritis Cartilage 2003 11 688 696 10.1016/S1063-4584(03)00149-3 12954240

[B32] Jung M Christgau S Lukoschek M Henriksen D Richter W Increased urinary concentration of collagen type II C-telopeptide fragments in patients with osteoarthritis Pathobiology 2004 71 70 76 10.1159/000074419 14707441

[B33] Carnemolla B Cutolo M Castellani P Balza E Raffanti S Zardi L Characterization of synovial fluid fibronectin from patients with rheumatic inflammatory diseases and healthy subjects Arthritis Rheum 1984 27 913 921 10.1002/art.1780270811 6466396

[B34] Jones KL Brown M Ali SY Brown RA An immunohistochemical study of fibronectin in human osteoarthritic and disease-free articular cartilage Ann Rheum Dis 1987 46 809 815 10.1136/ard.46.11.809 3322211 PMC1003397

[B35] Brown RA Jones KL Fibronectin synthesis and release in normal and OA human cartilage Eur J Exp Musculoskel Res 1992 1 25 32

[B36] Garnero P Ayral X Rousseau JC Christgau S Sandell LJ Dougados M Delmas PD Uncoupling of type II collagen synthesis and degradation predicts progression of joint damage in patients with knee osteoarthritis Arthritis Rheum 2002 46 2613 2624 10.1002/art.10576 12384919

[B37] Felson DT Kim YJ The futility of current approaches to chondroprotection Arthritis Rheum 2007 56 1378 1383 10.1002/art.22526 17469094

[B38] Dieppe P Disease modification in osteoarthritis: are drugs the answer? Arthritis Rheum 2005 52 1956 1959 10.1002/art.21124 15986369

[B39] Xie DL Meyers R Homandberg GA Fibronectin fragments in osteoarthritic synovial fluid J Rheumatol 1992 19 1448 1452 1433014

[B40] Billinghurst RC Dahlberg L Ionescu M Reiner A Bourne R Rorabeck C Mitchell P Hambor J Diekmann O Tschesche H Chen J Van Wart H Poole AR Enhanced cleavage of type II collagen by collagenases in osteoarthritic articular cartilage J Clin Invest 1997 99 1534 1545 10.1172/JCI119316 9119997 PMC507973

[B41] Felice BR Chichester CO Barrach HJ Type II collagen peptide release from rabbit articular cartilage Ann NY Acad Sci 1999 878 590 593 10.1111/j.1749-6632.1999.tb07736.x 10415782

[B42] Klatt AR Paul-Klausch B Klinger G Kühn G Renno JH Banerjee M Malchau G Wielckens K A critical role for collagen II in cartilage matrix degradation: collagen II induces pro-inflammatory cytokines and MMPs in primary human chondrocytes J Orthop Res 2009 27 65 70 10.1002/jor.20716 18655132

[B43] Wu JJ Lark MW Chun LE Eyre DR Sites of stromelysin cleavage in collagen types II, IX, X, and XI of cartilage J Biol Chem 1991 266 5625 5628 2005102

[B44] Chowdhury TT Bader DL Shelton JC Lee DA Temporal regulation of chondrocyte metabolism in agarose constructs subjected to dynamic compression Arch Biochem Biophys 2003 417 105 111 10.1016/S0003-9861(03)00340-0 12921786

[B45] Jeffrey JE Thomson LA Aspden RM Matrix loss and synthesis following a single impact load on articular cartilage in vitro Biochim Biophys Acta 1997 1334 223 232 9101717 10.1016/s0304-4165(96)00097-9

[B46] Valhmu WB Stazzone EJ Bachrach NM Saed-Nejad F Fischer SG Mow VC Ratcliffe A Load-controlled compression of articular cartilage inducesa transient stimulation of aggrecan gene expression Arch Biochem Biophys 1998 353 29 36 10.1006/abbi.1998.0633 9578597

[B47] Ragan PM Badger AM Cook M Chin VI Gowen M Grodzinsky AJ Lark MW Down-regulation of chondrocyte aggrecan and type-II collagen gene expression correlates with increases in static compression magnitude and duration J Orthop Res 1999 17 836 842 10.1002/jor.1100170608 10632450

[B48] De Croos JN Dhaliwal SS Grynpas MD Pilliar RM Kandel RA Cyclic compressive mechanical stimulation induces sequential catabolic and anabolic gene changes in chondrocytes resulting in increased extracellular matrix accumulation Matrix Biol 2006 25 323 331 10.1016/j.matbio.2006.03.005 16697175

[B49] Blain EJ Mechanical regulation of MMPs Front Biosci 2007 12 507 527 10.2741/2078 17127313

[B50] Blain EJ Mason DJ Duance VC The effect of cyclical compressive loading on gene expression in articular cartilage Biorheology 2003 40 111 117 12454394

[B51] Waldman SD Couto DC Grynpas MD Pilliar RM Kandel RA A single application of cyclic loading can accelerate matrix deposition and enhance the properties of tissue-engineered cartilage Osteoarthritis Cartilage 2006 14 323 330 10.1016/j.joca.2005.10.007 16324852

[B52] Kisiday JD Jin MS DiMicco MA Kurz B Grodzinsky AJ Effects of dynamic compressive loading on chondrocyte biosynthesis in self assembling peptide scaffolds J Biomech 2004 37 595 604 10.1016/j.jbiomech.2003.10.005 15046988

[B53] Wong M Siegrist M Cao X Cyclic compression of articular cartilage explants is associated with progressive consolidation and altered expression pattern of extracellular matrix proteins Matrix Biol 1999 18 391 399 10.1016/S0945-053X(99)00029-3 10517186

[B54] Kisiday JD Lee JH Siparsky PN Frisbie DD Flannery CR Sandy JD Grodzinsky AJ Catabolic responses of chondrocyte-seeded peptide hydrogel to dynamic compression Ann Biomed Eng 2009 37 1368 1375 10.1007/s10439-009-9699-9 19415495

[B55] Steinmeyer J Ackermann B The effect of continuously applied cyclic mechanical loading on the fibronectin metabolism of articular cartilage explants Res Exp Med (Berl) 1999 198 247 260 10.1007/s004330050108 10209760

[B56] Bonassar LJ Grodzinsky AJ Srinivasan A Davila SG Trippel SB Mechanical and physicochemical regulation of the action of insulin-like growth factor-I on articular cartilage Arch Biochem Biophys 2000 379 57 63 10.1006/abbi.2000.1820 10864441

[B57] Mauck RL Nicoll SB Seyhan SL Ateshian GA Hung CT Synergistic action of growth factors and dynamic loading for articular cartilage tissue engineering Tissue Eng 2003 9 597 611 10.1089/107632703768247304 13678439

[B58] Ramage L Nuki G Salter DM Signalling cascades in mechanotransduction: cell-matrix interactions and mechanical loading Scand J Med Sci Sports 2009 19 457 469 10.1111/j.1600-0838.2009.00912.x 19538538

[B59] Vincent TL McLean CJ Full LE Peston D Saklatvala J FGF-2 is bound to perlecan in the pericellular matrix of articular cartilage, where it acts as a chondrocyte mechanotransducer Osteoarthritis Cartilage 2007 15 752 763 10.1016/j.joca.2007.01.021 17368052

[B60] Millward-Sadler SJ Salter DM Integrin-dependent signal cascades in chondrocyte mechanotransduction Ann Biomed Eng 2004 32 435 446 10.1023/B:ABME.0000017538.72511.48 15095818

[B61] Homandberg GA Costa V Wen C Anti-Sense oligonucleotides to the alpha5 integrin subunit suppress cartilage chondrolytic activities of amino-terminal fibronectin fragments Osteoarthritis Cartilage 2001 10 381 393 10.1053/joca.2002.0524

[B62] Fitzgerald JB Jin M Dean D Wood DJ Zheng MH Grodzinsky AJ Mechanical compression of cartilage explants induces multiple time-dependent gene expression patterns and involves intracellular calcium and cyclic AMP J Biol Chem 2004 279 19502 19511 10.1074/jbc.M400437200 14960571

[B63] Kurz B Lemke A Kehn M Domm C Patwari P Frank EH Grodzinsky AJ Schünke M Influence of tissue maturation and antioxidants on the apoptotic response of articular cartilage after injurious compression Arthritis Rheum 2004 50 123 130 10.1002/art.11438 14730608

[B64] Henrotin YE Bruckner P Pujol JP The role of reactive oxygen species in homeostasis and degradation of cartilage Osteoarthritis Cartilage 2003 11 747 755 10.1016/S1063-4584(03)00150-X 13129694

[B65] Chen CT Bhargava M Lin PM Torzilli PA Time, stress, and location dependent chondrocyte death and collagen damage in cyclically loaded articular cartilage J Orthop Res 2003 21 888 898 10.1016/S0736-0266(03)00050-0 12919878

